# Estimating progression of Alzheimer’s disease with extracellular vesicle-related multi-omics risk models

**DOI:** 10.3389/fnagi.2025.1617611

**Published:** 2025-07-24

**Authors:** Xiao Zhang, Sanoji Wijenayake, Shakhawat Hossain, Qian Liu

**Affiliations:** ^1^Max Rady College of Medicine, University of Manitoba, Winnipeg, MB, Canada; ^2^Department of Applied Computer Science, University of Winnipeg, Winnipeg, MB, Canada; ^3^Department of Biology, University of Winnipeg, Winnipeg, MB, Canada; ^4^Department of Mathematics and Statistics, University of Winnipeg, Winnipeg, MB, Canada

**Keywords:** Alzheimer’s disease, multiomics, extracellular vesicles (EV), LASSO, Cox regression, biomarkers

## Abstract

**Background:**

Alzheimer’s Disease (AD) is heterogeneous and shows complex interconnected pathways at various biological levels. Risk scores contribute greatly to disease prognosis and biomarker discovery but typically represent generic risk factors. However, large-scale multi-omics data can generate individualized risk factors. Filtering these risk factors with brain-derived extracellular vesicles (EVs) could yield key pathologic pathways and vesicular vehicles for treatment delivery.

**Methods:**

A list of 460 EV-related genes was curated from brain tissue samples in the ExoCarta database. This list was used to select genes from transcriptomics, proteomics, and DNA methylation data. Significant risk factors included demographic features (age, sex) and genes significant for progression in transcriptomics data. These genes were selected using Cox regression, aided by the Least Absolute Shrinkage and Selection Operator (LASSO), and were used to construct three risk models at different omics levels. Gene signatures from the significant risk factors were used as biomarkers for further evaluation, including gene set enrichment analysis (GSEA) and drug perturbation analysis.

**Results:**

Nine EV-related genes were identified as significant risk factors. All three risk models predicted high/low risk groups with significant separation in Kaplan-Meier analysis. Training the transcriptomics risk models on EV-related genes yielded better AD classification results than using all genes in an independent dataset. GSEA revealed Mitophagy and several other significant pathways related to AD. Four drugs showed therapeutic potential to target the identified risk factors based on Connectivity Map analysis.

**Conclusion:**

The proposed risk score model demonstrates a novel approach to AD using EV-related large-scale multi-omics data. Potential biomarkers and pathways related to AD were identified for further investigation. Drug candidates were identified for further evaluation in biological experiments, potentially transported to targeted tissues via bioengineered EVs.

## Highlights

• The use of EV-related genetic risk factors for AD prognosis produced more accurate risk models when compared to using generic risk factors.
• Evaluation of significant EV-related risk factors revealed mitophagy as a relevant pathway and penfluridol as a potential repurposed treatment that can be used to treat AD.
• EV-related multi-omics data integration allows for a more comprehensive characterization of AD across biological layers.

## 1 Introduction

Aging is a natural process that affects all living organisms, but brings increased susceptibility to neurodegenerative disorders, such as Alzheimer’s Disease (AD). One in 10 people over the age of 65 is diagnosed with AD (Hou et al., 2019). As the global elderly population increases—with countries like the United States projecting an increase in the old-age dependency ratio from 28 (in 2020) to 41 (in 2060)—a significant burden on the healthcare infrastructure is forthcoming (Vespa et al., 2020). The increased prevalence of AD will increase financial pressure on the healthcare system, insurance services, personal care homes, and individual families (Azam et al., 2021). However, given the widespread impact of neurodegenerative disorders, targeting AD through treatment and prevention may offer the most impactful improvements in quality of life worldwide.

AD is a type of dementia which interferes with cognition and impacts the quality of life. Different pathological mechanisms have been proposed to cause AD, but we lack a clear understanding of the full mechanism. However, a common denominator of AD is the presence of neuritic plaques, neurofibrillary tangles (NFTs), and cortical neuronal degeneration (Kumar et al., 2024). Neuritic plaques are formed by amyloid beta (Aβ) peptides (Kumar et al., 2024). Neurofibrillary tangles are formed by tau protein in neurons, which have a higher chance of being misfolded when in the phosphorylated state (p-tau) (Kumar et al., 2024). Previous studies have shown that mutations in candidate genes, mainly APP, PSEN1 & 2, ADAM10, ADAM1J, APOE, are associated with neurodegeneration (Neuner et al., 2020). Recently, extracellular vesicles have emerged as significant contributors to AD pathogenesis and offer a very unique, yet underused, avenue for improving not only AD treatment, but AD prognosis (Sarko and McKinney, 2017). In the context of neurodegeneration, small EVs ranging from 50 to 150 nm that are derived from invagination of the late endosome, often referred to as “exosomes,” have garnered attention as of lately. Brain-derived small EVs transport misfolded proteins like Aβ, p-tau, and alpha-synuclein, contributing to the spread of pathological processes across brain regions (Sarko and McKinney, 2017). Furthermore, brain-derived small EVs are thought to cross the blood-brain barrier, to and from the peripheral circulation (Li et al., 2019). This enables us to measure small EVs in peripheral circulation as biomarkers for AD diagnosis and highlights their therapeutic potential in drug delivery to the brain to target AD (Fayazi et al., 2021). However, the current knowledge about EVs provides few methods (e.g., surface markers) that can distinguish blood- and brain-derived EVs with moderate sensitivity and specificity. L1 cell adhesion molecule (L1CAM) is a common biomarker for identifying brain-derived EVs, but it is also present in blood-derived EVs (Bravo-Miana et al., 2024). Alternatives, such as glutamate aspartate transporter (GLAST) and myelin oligodendrocyte glycoprotein (MOG), provide more sensitive and specific detection of brain-derived EVs, but only covers a small range of possible EVs originating from the CNS (Bravo-Miana et al., 2024).

The advent of high-throughput sequencing (HTS) and automated processing pipelines allowed large quantities of multi-omics data to be collected. While human interpretation generally only looks at one layer of multi-omics data, algorithms can process multiple layers of omics data to provide a more comprehensive view of interconnected pathways. Three key types of data include transcriptomics, DNA methylation, and proteomics, which help pinpoint several hallmarks of aging. At the DNA level, DNA methylation data considers effects of *Epigenetic alterations* (Azam et al., 2021). At the mRNA level, transcriptomics profiling reveals *Genomic instability and DNA damage* and *Telomere attrition* (Azam et al., 2021). At the protein level, proteomics data reveals *Loss of proteostasis* (Azam et al., 2021). Thus, EV-related multi-omics information may prove to be valuable in exploring the various pathologic mechanisms and potential therapeutic targets of neurodegenerative disorders. EV-related biomarkers have been successfully used to construct risk models for triple-negative breast cancer (Qiu et al., 2021). However, there are no EV-related risk models for neurodegenerative disorders.

The idea of using risk models has been seen in predicting complex polygenic chronic diseases such as diabetes mellitus (Davies et al., 2017) and depression (Pearson-Fuhrhop et al., 2014). Beyond measuring patient disease risk, significant risk factors identified may be further investigated as potential biomarkers or therapeutic targets. This can provide a set of prognostic tools and therapeutic targets that can help with disease treatment. More recently, Qiu et al. developed risk models for breast cancer (BC) using EV-related genomics data. This study revealed the importance of exosomes and other EVs in contemporary diseases and reinforces the potential of EVs to influence AD (Garcia-Contreras and Thakor, 2023).

Often, risk scores are calculated based on generic risk factors, such as demographics (age, sex, etc.) and basic clinical assessments (blood pressure, cognitive ability, depression, etc.) (Anstey et al., 2021). While these are easily collected and readily available, there lacks an individuality to the resulting risk score. Technological improvements of the past decade allowed us to gather vast amounts of personalized data at various levels of biology, such as genetic and epigenetic, RNA, microRNA, and protein levels (Wang et al., 2024). Alternatively, risk models that do not focus on generic risk factors instead utilize survival analysis on diseases with a clear progression path (often defining the final event as death) or create polygenic risk scores based on genome-wide association studies (GWAS). This ends up prioritizing various diseases with high fatality rates through traditional survival analysis or utilizes too broad of a dataset in GWAS-based polygenic risk scores.

Recently, researchers have constructed large perturbational drug datasets, which we can use to control the expression of candidate genes that are associated with disease phenotypes. Drugs identified from a large perturbational dataset, such as Connectivity Map (CMap), which highly disrupt certain gene signatures in the identified biomarkers can be further evaluated as treatments (Subramanian et al., 2017). These drugs can potentially be bioengineered within EVs to enhance transport across complex biological barriers, and reduce biphasic release and instability issues that are common in synthetic nanovesicles and nanoparticles. The natural occurrence of EVs in our body results in reduced clearance by our immune system, when compared to other synthetic molecules. The possible addition of fusion proteins also allows for highly targeted release at specific binding sites (Nowak et al., 2023). Similarly, gene set enrichment analysis (GSEA) can generalize multiple genes into various permutations of unique biological pathways using *a priori* gene sets curated by subject-matter experts. Enrichr is a web interface developed at the Ma’ayan Laboratory which enables GSEA to be performed on 35 different gene set libraries (Chen et al., 2013; Kuleshov et al., 2016; Xie et al., 2021). The use of GSEA for humans rose to prominence following the completion of the Human Genome Project. Gene sets, such as the Kyoto Encyclopedia of Genes and Genomes (KEGG), regularly updated and revised to include new discoveries (Kanehisa and Goto, 2000).

The proposed EV-related risk model for AD aims to incorporate the best of technologies used in previous studies and apply them to neurodegenerative disorders. It improves upon generic risk factors by using large-scale multi-omics data that is unique to each individual patient. The broad scope of polygenic risk scores is also solved by our EV-focused approach to genes in the selected multi-omics data. Conventional survival analysis in Cox regression typically applies to disease processes which have distinct changes in status from a healthy to diseased state. This is replaced with time-to-event (TTE) analysis based on progression-free status (PFS), which measures time from initial suspicion (e.g., undiagnosed symptoms) to disease progression. The modified approach accommodates lower mortality rates observed in neurodegenerative disorders. Further analysis of identified pathways using GSEA and CMap will provide insight into future directions.

## 2 Materials and methods

**FIGURE 1:**
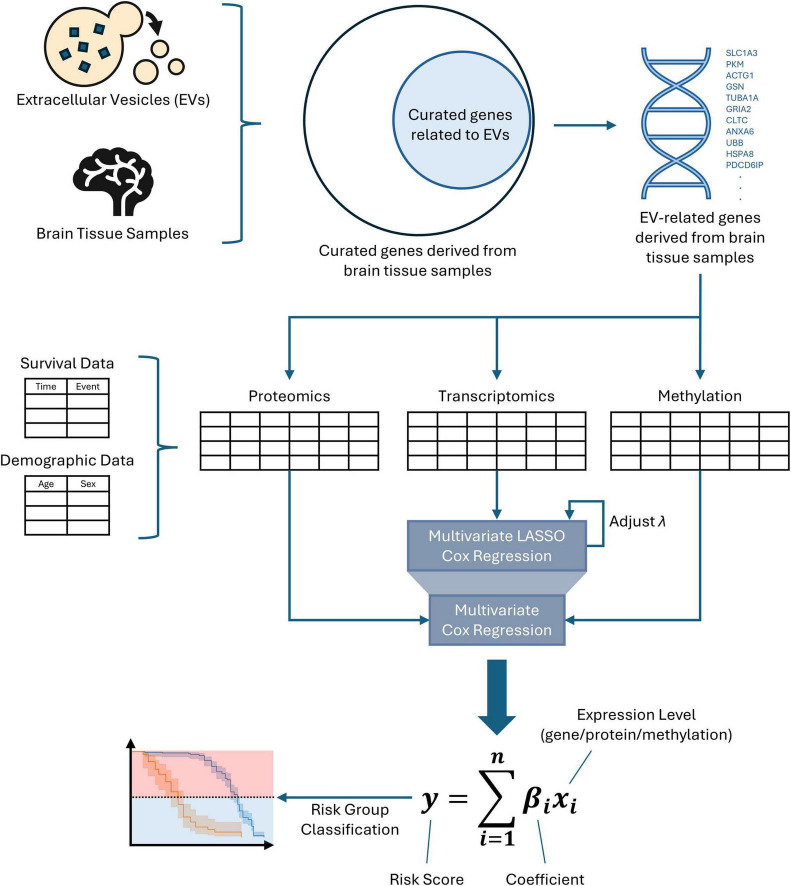
Risk model construction workflow. A list of EV-related genes derived from brain tissue samples is used to filter DNA methylation, transcriptomics, and proteomics datasets. Significant gene signatures are selected using multivariable Cox regression with LASSO regularization and combined with demographic data to identify significant risk factors. Scaling coefficients for these risk factors are then calculated using multivariable Cox regression.

The overall workflow for risk model construction is shown in Figure 1. A list of EV-related genes derived from brain tissue is curated. After filtering multi-omics data using the list of EV-related genes, this downscaled the data to focus on brain-derived EV-related genes. Risk models were constructed for each type of omics data using the Cox proportional hazards model. The initial iteration identified significant covariates, which were then combined with demographic data to produce a list of significant risk factors. The second iteration calculated coefficients for each significant risk factor. These coefficients were used with the respective data type to calculate risk score. The risk score allowed patients to be classified as high- or low-risk, and the significant risk factors were further investigated as potential biomarkers and/or therapeutic targets. The source code of the workflow is available at https://github.com/maomao853/AD-Multi-Omics-EV-Risk-Model.

### 2.1 Data sources

#### 2.1.1 Extracellular vesicles

A list of genes was curated from ExoCarta (Keerthikumar et al., 2016), derived from small EVs isolated from brain tissue samples. Data from isolated EVs were obtained from previously published studies, culminating in the repository hosted on ExoCarta (Keerthikumar et al., 2016). This yielded 356 unique brain-derived EV-related genes (Supplementary Data 1) from small EVs across five cell types: cortical neurons, microglia, Mov neuroglial cells, neural stem cells, and oligodendrocytes.

#### 2.1.2 Multi-omics

Transcriptomics and DNA methylation data was obtained from the Alzheimer’s Disease Neuroimaging Initiative (ADNI) project (Petersen et al., 2010). Proteomics data were obtained from UK Biobank (Sudlow et al., 2015). Evaluation data for gene expression were obtained from the Gene Expression Omnibus (GEO), specifically study GSE5281 (Liang et al., 2007; Liang et al., 2008a; Liang et al., 2008b; Readhead et al., 2018). Table 1 shows the demographics and disease status of the patients included in this study.

**TABLE 1:** Clinical and demographic characteristics of patients in studies used.

Dataset	Age		Sex			Disease status		
	Median	SD	Male	Female	Unknown	CN	MCI	AD
UK Biobank	72.46	8.12	228,990	273,177	0	494,389	3,273	4,476
ADNI	72.65	8.05	1,489	1,429	21	952	1,288	480
GSE5281	78.83	10.07	103	57	0	74	0	87

Disease status measures three major stages of AD progression: cognitively normal (CN), mild cognitive impairment (MCI), and AD.

In ADNI data, patient records were converted to TTE data. The event was measured as the diagnosis of AD, which had a Boolean value (0 for non-AD, 1 for AD). Time was measured as the duration from the initial visit to the first occurrence of the event, or to the last follow-up if the event did not occur. If the event occurred immediately, then time was zero. Similarly, in UK Biobank data, patient records were converted to TTE data. The event was based on ICD-10 classifications (World Health Organization, 2004), measured as the diagnosis of G30 (AD). Survival time was measured from the initial diagnosis of G31 or G32 (other degenerative diseases/disorders of nervous system), until the event occurs, or until the last follow-up if the event did not occur.

### 2.2 Pre-processing

#### 2.2.1 DNA methylation

DNA methylation data were retrieved and pre-processed using the *minfi* package in R (Aryee et al., 2014). The raw probe-level methylation data were converted to gene-level data using the included annotation information using Supplementary Equation 1. Duplicated genes were aggregated using the median of their values, reducing the initial 865,859 probe loci to 66,069 genes. Filtering brain-derived EV-related genes based on our curated list further reduced the number of genes from 66,069 to 248. This data was combined with patient survival and demographics information by matching their RID (roster ID) and reduced the sample size from 1,905 to 649.

#### 2.2.2 Transcriptomics

Transcriptomics data were filtered based on brain-derived EV-related genes and aggregated using the average of their values. This resulted in a gene set size reduction from 48,157 to 313 and sample size reduction from 744 to 142.

#### 2.2.3 Proteomics

Proteomics data was selected and exported manually from UK Biobank using the Research Analysis Platform (RAP). Only brain-derived EV-related genes were selected and filtered based on neurodegenerative diseases defined by ICD-10 (World Health Organization, 2004). After filtering, the gene set size was reduced from 1,463 to 91 and sample size was reduced from 54,306 to 39. Missing values were imputed using the KNNImputer (*k* = 2, uniform weights) from scikit-learn (Pedregosa et al., 2011). KNN imputation estimates missing values based on the *k*-nearest neighbors in the training set, which provides easy implementation and high accuracy, just behind lowest of detection (LOD) and random drawing from a left-censored normal distribution (ND) (Jin et al., 2021). The parameter *k* indicates the number of neighbors to consider and *uniform weights* assigns all neighborhood points equal weights.

### 2.3 Risk score

The risk score for each dataset is modeled using Equation 1. This linear function provides a transparent view of how each significant risk factor impacts the risk score.


(1) $$y=\sum_{i=1}^{p}\beta_{i}\,x_{i}$$

Where *y* is the risk score, *x* represents the gene/protein/methylation expression level, and β represents the scaling coefficient associated with *x*.

A subset of gene expression data, filtered using the curated list of EV-related genes localized in brain tissue, was used to train the transcriptomics risk model (Equation 2).


(2) $$\lambda \left(t|x_{i}\right)=\lambda_{0}\left(t\right)\times e^{x_{i}^{T}\,\beta }$$

Where *t* represents time, *x_i_* represents the covariate matrix for the subject *i*, and β represents the scaling coefficients for the covariate matrix. The baseline hazard λ_0_(*t*) remains constant between different subjects.

The Cox model associates covariates with TTE information. The Least Absolute Shrinkage and Selection Operator (LASSO) regularization filters out insignificant covariates by optimizing the model coefficients and maximizing sparsity. We minimize the log-partial likelihood subject to an L1 regularization λ(||β||). This constraint shrinks coefficients (β) toward zero, resulting in some coefficients being exactly zero. This approach yields a more interpretable final model. We used five-fold cross-validation and measured concordance index (C-index), outlined in Supplementary Equation 2, to select the best λ value, which yielded the best model performance.

Significant genes (*p* < 0.05) identified from a multivariable Cox regression model with LASSO, based on transcriptomics data, were combined with patient demographics (age and sex) to establish the key covariates in the transcriptomics risk model. This model was then used in a second multivariable Cox model (without LASSO) to refine the significant covariates and construct the final transcriptomics risk model. These significant covariates were subsequently evaluated using DNA methylation and proteomics data. Gene signatures and demographic data were filtered based on these significant covariates in the transcriptomics risk model, and scaling coefficients were determined using multivariable Cox regression (without LASSO). This resulted in two additional risk models, one each for DNA methylation and proteomics.

### 2.4 Evaluation

Three risk models (transcriptomics, methylation, and proteomics) were used to calculate individual risk scores for their respective cohort in the ADNI or UK Biobank studies. Each cohort was divided into high- and low-risk groups based on the median risk score. Difference in PFS was visualized using Kaplan-Meier (KM) plots and quantified using log-rank tests. Gene expression data from GEO were also used as external datasets to evaluate the potential biomarkers.

Comparison risk models were constructed through a similar process, but using the entire cohort’s gene set instead of the EV-related gene list. Before entering the previously described LASSO Cox regression, the gene list underwent preliminary filtering: variance thresholding and univariate Cox regression. Variance filtering, using VarianceThreshold in scikit-learn (Pedregosa et al., 2011), removed genes with values of one or zero in more than 70% of samples. Each remaining gene underwent univariate Cox regression; significant genes (*p* < 0.05) were combined with demographics data (age and sex) to create the final list of risk factors. This list was then used in the original risk model construction pipeline for transcriptomics data. Kaplan-Meier (KM) plots were compared for all three data types, and classification accuracy was measured using external GEO datasets.

GSEA was performed on the set of genetic risk factors using Enrichr (Chen et al., 2013; Kuleshov et al., 2016; Xie et al., 2021), providing insights into pathways implicated in AD. These genes were also evaluated in the CMap perturbational dataset (Subramanian et al., 2017). The CMap dataset contains Connectivity Scores, which compares effects of *query* and *reference* molecules on specific genes. This score combines the nominal *p*-value, false discovery rate (FDR), and Tau (τ), a metric comparing an observed enrichment score to all others in the database. This score ranges from -100 (representing opposing effects) to +100 (representing similar effects) (Subramanian et al., 2017). Potential therapeutic agents were identified by selecting perturbagens/drugs with < -90 or > 90 connectivity score for the significant genes and evaluating their z-scores for disruption of regular gene functions (>1.96 or < −1.96).

## 3 Results

An EV-focused approach to estimating AD progression produced three key equations (Equations 3–5) to calculate individual risk scores. Missing features in Equations 4, 5 resulted from differences in datasets and zero coefficients when isolating for significant risk factors.


(3) $$\,Ri\,s\,k\,S\,c\,o\,r\,e_{t\,r\,a\,n\,s\,c\,r\,i\,p\,t\,o\,m\,i\,c\,s}=\left(-0.20\times C\,C\,T\,8\right)+$$

(3.41×H⁢I⁢S⁢T⁢1⁢H⁢3⁢A)+(0.76×H⁢I⁢S⁢T⁢1⁢H⁢4⁢F)

+(0.66×H⁢T⁢R⁢A⁢1)+(-3.42×K⁢R⁢T⁢14)

+(-0.99×K⁢R⁢T⁢5)+(-2.03×N⁢A⁢R⁢S)+

(2.09×R⁢A⁢B⁢5⁢C)+(-0.71×U⁢B⁢B)+(0.37×S⁢e⁢x)

(4) $$\,Ri\,s\,k\,S\,c\,o\,r\,e_{p\,r\,o\,t\,e\,o\,m\,i\,c\,s}=\left(1.80\times K\,R\,T\,14\right)+$$

(0.42×K⁢R⁢T⁢5)+(-0.01×S⁢e⁢x)+(0.05×A⁢g⁢e)

(5) $$R\,i\,s\,k\,S\,c\,o\,r\,e_{m\,e\,t\,h\,y\,l\,a\,t\,i\,o\,n}=\left(34.18\times C\,C\,T\,8\right)+\left(1.93\times H\,I\,S\,T\,1\,H\,3\,A\right)$$

+(-15.35×H⁢I⁢S⁢T⁢1⁢H⁢4⁢F)+(-26.56×H⁢T⁢R⁢A⁢1)+

(15.63×K⁢R⁢T⁢14)+(-2.48×K⁢R⁢T⁢5)+(-3.38×N⁢A⁢R⁢S)+

(-1.92×U⁢B⁢B)+(0.37×S⁢e⁢x)

**FIGURE 2:**
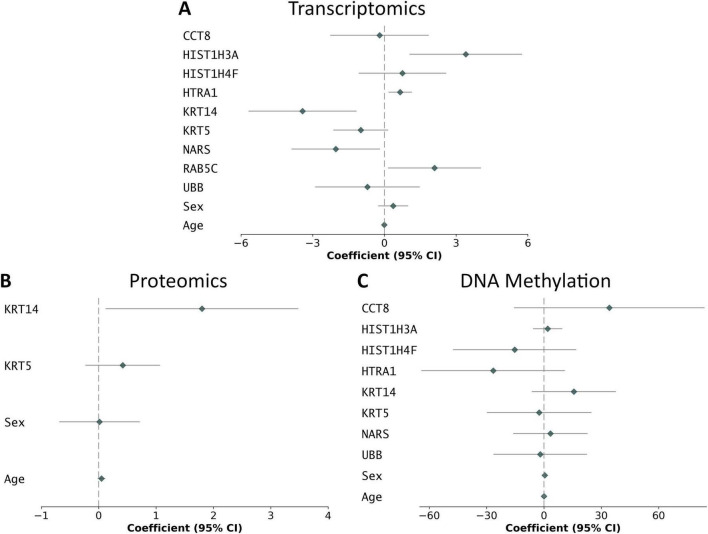
Forest plot of scaling coefficients for EV-related AD risk model covariates. Scaling coefficients are modeled using DNA methylation, transcriptomics, and proteomics data. Covariates include demographic features and gene signatures (*p* < 0.05) selected from multivariable LASSO Cox regression. Coefficient values are calculated using multivariable Cox regression. **(A)** Transcriptomics, **(B)** proteomics, and **(C)** methylation.

Forest plots (Figure 2) visualized the effect of each gene and its associated scaling coefficient. Genes exhibited higher variance than demographic features across all three risk models. Age had a zero coefficient in transcriptomics and methylation risk models (both from the ADNI dataset), but a non-zero coefficient in the proteomics risk model (from the UK Biobank dataset). Overall, demographic features had less impact than the gene signatures identified by TTE analysis. At the DNA level, our methylation risk score showed CCT8 had the largest association with AD prognosis, based on the DNA methylation risk score. At the mRNA level, HIST1H3A had the largest association with AD prognosis, based on the transcriptomics risk score. At the protein level, KRT14 had the largest association with AD prognosis, based on the proteomics risk score.

**FIGURE 3:**
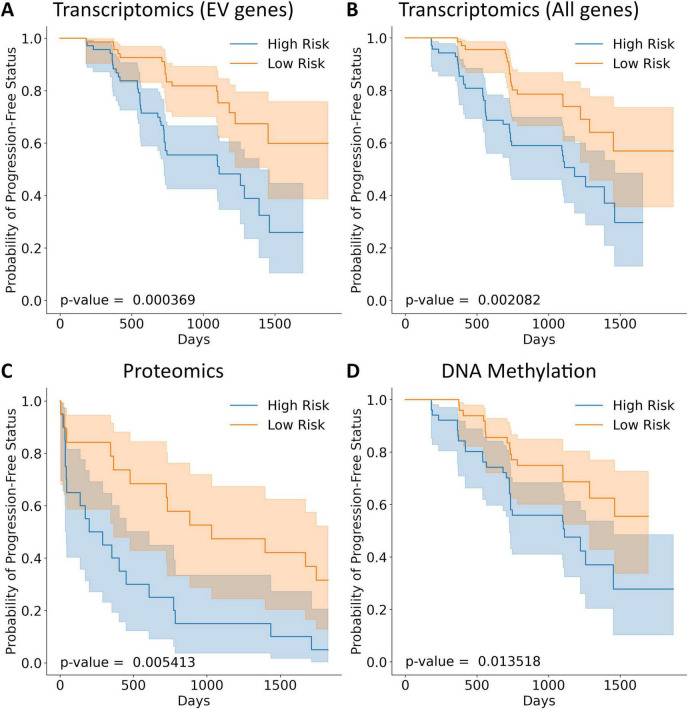
Kaplan–Meier plots for difference in progression-free status between high and low risk individuals. Events were observed over a span of 5 years. Risk groups were separated by a median risk score cutoff. Kaplan–Meier analysis was performed on the transcriptomics model trained on two subsets of data: **(A)** transcriptomics model for EV-related genes, **(B)** transcriptomics model for all genes, **(C)** proteomics model for EV-related genes, and **(D)** DNA Methylation model for EV-related genes.

Three risk models—derived from transcriptomics, proteomics, and methylation data—significantly separated high- and low-risk groups within their respective cohorts (ADNI and UK Biobank). KM curves of TTE analyses showed that the low-risk group had a significantly higher probability of PFS than the high-risk group (Figure 3). Log-rank tests confirmed significant separation for all models, with the transcriptomics model demonstrating the greatest separation of high- and low-risk groups, followed by the proteomics and methylation models.

Figure 3 shows that the low-risk group consistently yields better PFS than the high-risk group. The separation between the curves and the low *p*-values confirms this statistical significance. The magnitude of the difference varies across the analyses. Some analyses show a larger difference in PFS than others. For example, Figure 3C demonstrates that patients classified as high-risk based on their proteomic profiles have a significantly lower probability of remaining progression-free over time compared to those classified as low-risk. The proteomic markers appear to successfully stratify patients into groups with differing prognoses.

**FIGURE 4:**
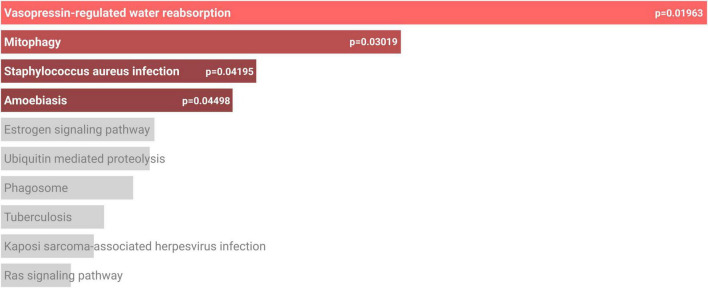
Pathways of genetic risk factors in the EV-related AD risk model. Genes were evaluated together on the KEGG database using Enrichr (Chen et al., 2013; Kuleshov et al., 2016; Xie et al., 2021). Four pathways were significant with *p* < 0.05.

The EV-related genes resulted in significant separation of high- and low-risk groups, indicated by a lower p-value in the logrank test. A variety of normal and skewed distributions were observed for risk scores (Supplementary Figure 1). When used for prediction and classification tasks on the GSE5281 dataset, the risk model targeting EV-related genes showed a 28% increase in accuracy, a 26% increase in F1-score, and a 35% increase in ROC AUC score when compared to the unfiltered risk model Figure 4.

**FIGURE 5:**
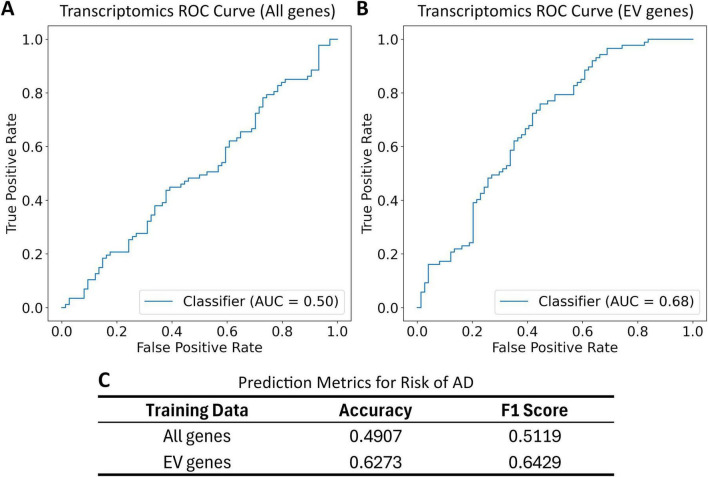
Receiver operating characteristic (ROC) curve and classification metrics for transcriptomics risk model applied to the GSE5281 dataset. Patients are labeled as cognitively normal (CN) or having Alzheimer’s Disease (AD). These labels are compared to risk group classification based on a risk score and a median risk score cutoff. All gene list includes all genes in the transcriptomics dataset. EV gene list includes all overlapping genes in the transcriptomics dataset and EV gene list. **(A)** The ROC for the transcriptomics risk model trained on all genes; **(B)** the ROC curve for transcriptomics risk model trained on EV-related genes; **(C)** the prediction metrics for transcriptomics risk model trained on all genes and EV-related genes.

GSEA of genetic risk factors showed significance in the pathways of vasopressin-regulated water reabsorption, mitophagy, *Staphylococcus aureus* infection, and amoebiasis Figure 5. Red bars in indicate significance (*p* < 0.05), with bar length inversely proportional to the *p*-value.

**FIGURE 6:**
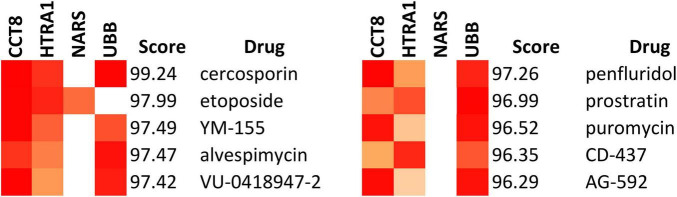
Drugs connected to significant genetic risk factors. Impact on genes is assessed based on connectivity score in CMap; significance is indicated by a connectivity score <−90 or >90. Ten drugs showed significant connectivity score for the genes CCT8 and HTRA1. Nine drugs showed significant connectivity score for the gene UBB. One drug showed a significant connectivity score for the gene NARS.

Evaluation on CMap showed several drugs that are highly connected with the genes CCT8, HTRA1, NARS, and UBB Figure 6. The drugs in contains several experimental drugs and only one FDA-approved drug — etoposide.

Further analysis of the identified drugs and target genes revealed the following dose and durations that provide optimal perturbation of the targeted genes (Table 2). Perturbation, measured as the *z*-score of cell line survival disturbance, was significant for all experimental drugs (AG-592, cercosporin, penfluridol, and puromycin), none of which are currently FDA-approved.

**TABLE 2:** List of drugs targeting significant genes in the AD risk model. Significant perturbation levels are measured as a *z*-score of less than -1.96 or greater than 1.96.

Drug	Role	Gene	Dose (μM)	Time (h)	*z*-score
AG-592	Tyrosine kinase inhibitor	CCT8	10	24	2.63
AG-592	Tyrosine kinase inhibitor	NARS	10	24	2.59
AG-592	Tyrosine kinase inhibitor	UBB	10	6	2.44
Cercosporin	Protein kinase C inhibitor	CCT8	10	24	2.76
Cercosporin	Protein kinase C inhibitor	HTRA1	10	6	4.52
Cercosporin	Protein kinase C inhibitor	NARS	10	24	2.42
Cercosporin	Protein kinase C inhibitor	UBB	10	24	2.09
Penfluridol	Dopamine receptor antagonist	CCT8	30	6	-5.76
Penfluridol	Dopamine receptor antagonist	HTRA1	30	6	7.29
Penfluridol	Dopamine receptor antagonist	NARS	30	24	-9.77
Penfluridol	Dopamine receptor antagonist	UBB	30	6	3.91
Puromycin	Adenosine receptor agonist	CCT8	10	24	3.48
Puromycin	Adenosine receptor agonist	HTRA1	10	24	4.65
Puromycin	Adenosine receptor agonist	NARS	10	24	3.83
Puromycin	Adenosine receptor agonist	UBB	10	24	2.87

## 4 Discussion

In general, the proposed risk model for AD produced a significant separation of high- and low-risk individuals, as evidenced by significant log-rank tests (*p* < 0.05) (Figure 3). Genes identified in various omics data (CCT8, HIST1H3A, HIST1H4F, HTRA1, KRT14, KRT5, NARS, RAB5C, UBB) showed a stronger influence on the risk score than demographic factors. This underscores the advantage of using large multi-omics datasets generated by high-throughput screening methods over generic risk factors obtained from screening and clinical assessments. The reliance on older age cohorts in neurodegenerative disease research often introduces sampling biases, as seen in the ADNI and UK Biobank cohorts. By using factors that are less prone to bias, our risk model can incorporate more datasets, leading to more accurate risk estimations.

Comparing risk models developed with and without the EV-focused filtering (Figures 3, 5) shows the benefits of EV-focused filtering. The lower *p*-value of the model using EV-related genes indicates superior separation of risk groups compared to the model using all genes. Further evaluation in GSE5281 (Liang et al., 2007; Liang et al., 2008a; Liang et al., 2008b; Readhead et al., 2018) confirmed this improved predictive performance, reinforcing the value of an EV-focused approach to AD risk modeling. These results highlight the importance of EV-related genes as risk factors for AD.

Beyond predictive power, the identified genetic risk factors offer valuable targets for functional and enrichment analyses. Among the nine genetic risk factors, three genes of interest—CCT8, RAB5C, UBB—have functions relevant to AD. Additionally, there is a significant effect from the KRT gene family (i.e., KRT5 and KRT14). However, KRT genes are common contaminants, especially in proteomics studies. Thus, elevated KRT coefficients are likely from environmental or operator error. CCT8, a part of the CCT chaperonin family, plays a crucial role in protein folding and transport (Yang et al., 2018). Previous GWAS studies have implicated CCT8 in suppressing Aβ-induced AD (Khabirova et al., 2014). Mutations in CCT8 could theoretically increase the risk of Aβ misfolding, a key factor in AD pathogenesis. This extends to the epigenetic level, where DNA methylation can dynamically alter gene expression. Suppression of CCT8, an important chaperone in Aβ folding, poses significant risk, as reflected by its high scaling coefficient in the DNA methylation risk score (Equation 5). RAB5C, a member of the Rab family and Ras superfamily (Han et al., 1996), is another risk factor. This gene is integral to docking/fusion of vesicles (Barbera et al., 2019) through promotion of tethering proteins, which pull vesicles closer together, and SNARE structures, which further reduce vesicle distance and initiate fusion (Borchers et al., 2021). Previous studies have observed signs of Rab5 overactivation in post-mortem brain tissue from AD patients, dysregulating the endo-lysosomal system (Xu et al., 2018). This is the second most impactful gene in the transcriptomics risk score, which reflects the potential of the endosomal system in transporting misfolded proteins. The gene UBB is also a risk factor that provides insight for AD. Although normal UBB codes for ubiquitin B, an altered variant has been observed to accumulate in the brains of AD patients. Since ubiquitin is normally involved in protein cycling through proteolysis, a failure to break down damaged proteins can lead to the accumulation of misfolded proteins, as seen in AD (Maniv et al., 2023).

Mitophagy, selected from the four pathways (Figure 4), involves the regulation of mitochondrial degradation. This is a hallmark of aging and neurodegenerative disorders (Fang et al., 2019). Mitochondria are involved in energy production through key metabolic pathways. This rapid energy production cycle involves transfer of highly charged electrons, which may produce dangerous byproducts that must be eliminated by important mitochondrial pathways. Mitochondrial aging leads to dysfunctional pathways, energy deficits, and increased retention of dangerous byproducts, such as reactive oxygen species (ROS) (Spinelli and Haigis, 2018). These factors can contribute to the accumulation of Aβ and p-tau proteins, which are prominent drivers of AD (Kerr et al., 2017). Similarly, imbalances in mitochondrial fusion and fission could cause increased ROS generation via mtDNA mutation, defective mitochondria, or abnormally distributed mitochondria (Bonda et al., 2010). These effects drive the pathophysiology of many diseases. Therefore, reduction of mitophagy with aging (Wen et al., 2022) increases the risk of developing AD. Other pathways, such as vasopressin-regulated water reabsorption, staphylococcus aureus infection, and amoebiasis, were previously thought to have a loose connection with AD. Vasopressin-regulated water reabsorption has been thought to be a possible mechanism influencing the development of AD through decreased concentrations of vasopressin in CSF and brain tissue in patients with AD. Furthermore, the inability for patients with AD to respond to osmotic stimuli supports that vasopressin-regulated water reabsorption is significant in relation to AD and demonstrates the potential for vasopressin regulation to complement traditional treatment of AD (Norbiato et al., 1988). Staphylococcus infection as a significant pathway supports the possibility that human pathogens play a potential role in the development of AD. This process was proposed to be caused by increased cytokines and chemokines, which pass through the blood-brain barrier and triggers protein misfolding. The proposed cognitive improvement from antimicrobial drugs also supports further studies of staphylococcus infections as a contributor to AD pathogenesis (Catumbela et al., 2023). While amoebiasis has no previous links to AD, it has been seen to alter the gut microbiome (Ankri, 2021). The effect of the gut microbiome has been speculated as a modulator of AD, thus supporting the potential for the gut microbiome as a therapeutic target for management of AD (Catumbela et al., 2023).

Penfluridol emerged as the most impactful drug based on its z-scores affecting genes in our significant risk factors (Table 2). Previous studies suggest its potential to reduce AD severity. Although it is a potent neuroleptic drug used to treat psychotic disorders since the 1970s (Janssen et al., 1970), penfluridol also possesses antioxidative properties (Podsiedlik et al., 2022). Given that oxidizing agents contribute to Aβ accumulation, which is a principal hallmark of AD (Azam et al., 2021), the antioxidative effects of penfluridol can potentially be repurposed to treat AD. Puromycin is a protein synthesis inhibitor which caused significant perturbation to selected biomarkers. While its name is similar to puromycin-sensitive aminopeptidase (PSA), which has proven to slow down progression of AD by decreasing p-tau, it is puromycin that exhibits significant effects in analysis. As an antibiotic, puromycin kills pathogens, which is a proposed mechanism to reduce development of AD and improve cognitive ability in AD (Catumbela et al., 2023). Additionally, puromycin inhibits cholinesterase, which has shown to offset the destruction of cells that produce acetylcholine, maintains cholinergic transmission, and improves AD prognosis (Ahmed et al., 2022). However, the combined usage of PSA and puromycin needs caution, since puromycin is a selective inhibitor of natural PSA in the body (Reddi et al., 2020). Other drugs emerged from CMap which have statistical significance but no association with the treatment of AD. Cercosporin, a fungal toxin from the genus Cercospora, has no known therapeutic effects for AD. It was likely selected due to the usage of ROS in its mechanism of action (Newman and Townsend, 2016). Similarly, AG-592 is an experimental small molecule from the CMap dataset that acts as a tyrosine kinase inhibitor, which has no known effect on AD (Evangelista et al., 2022).

This study has some limitations due to data availability and standardization. Unlike clinical data for various cancers, AD lacks longitudinal studies required for TTE analysis. These studies are often separate from omics data collected from patients, requiring data collection and aggregation from different sources and assays with varying annotations. Consequently, non-standardized collection methods resulted in substantial unusable non-overlapping data.

Future research could explore aspects that are not covered in this study, such as biological validation, drug testing, and expansion to cover other neurodegenerative diseases. To evaluate the biomarkers identified in this study, animal models may be used with gene knockouts of the identified biomarkers to observe their effects on the status of AD. A similar process could be used to target the rate-determining step of a metabolic pathway. Drugs causing strong perturbations in the identified biomarkers could also be evaluated for their potential to reduce AD. Transgenic animal models expressing human amyloid precursor protein (APP) can reflect the efficacy of the proposed drugs. EVs can also be explored as transport vesicles that can cross the blood-brain barrier, facilitating drug delivery and biomarker detection via non-invasive testing (Pauwels et al., 2021). Once established, this workflow can be expanded to other neurodegenerative diseases to improve prognosis, identify new biomarkers, and identify potential treatments. Genomic biomarkers could be combined with radiological biomarkers extracted from medical images in future studies.

## 5 Conclusion

In conclusion, we demonstrated the effectiveness of EV-related multi-omics risk scores in predicting AD. The risk model, constructed using transcriptomics, proteomics, and DNA methylation data, successfully predicted high- and low-risk individuals based on significant risk factors. Nine genetic risk factors contributed substantially to prognosis, while demographic risk factors contributed much less. Three genetic risk factors were found to have functions highly relevant to AD, and enrichment analysis of all genetic risk factors identified the *Mitophagy* pathway as significant. Four drugs had significant connectivity with the genetic risk factors. Overall, this study established a foundation for future biological evaluation using the identified EV-related biomarkers and potential expansion to other neurodegenerative diseases.

## Data Availability

The original contributions presented in this study are included in this article/Supplementary material, further inquiries can be directed to the corresponding author.
