# Supplementary material for: Estimating progression of Alzheimer’s disease with extracellular vesicle-related multi-omics risk models
Source: Front Aging Neurosci. 2025 Jul 24;17:1617611. doi: 10.3389/fnagi.2025.1617611 (PMC12328399; doi:10.3389/fnagi.2025.1617611)
Supplement: Supplementary file 2 [file Supplementary_file_1.docx]

# Supplementary Text

## IDAT File Processing

DNA methylation data was retrieved in raw IDAT format and pre-processed using the *minfi* package in R [1]. The red and green intensity channels were converted into a GenomicRatio Set. Beta values (β) were used to represent methylation levels after the pre-processing steps.

Equation S1

$$\beta=\frac{M}{M+U+100}$$

Where $M$ and $U$ represent the methylated and unmethylated signal intensity respectively.

## Concordance Index

The concordance index (C-index) metric is used to evaluate performance of Cox regression. Our modified “time-to-event” (TTE) analysis uses similar *model* (M) and *time to event* (T) as variables being measured. Thus, we use the same generic C-index calculations.

Equation S2

$$C=P(M_{j}>M_{i}|T_{j}<T_{i})$$

Where $M$ is a model, also known as a map from the subject space, such that $M\mathbb{\in R}$. Observation time until event is represented by $T$, such that $T=T_{obs}$ if censoring does not occur, otherwise $T>T_{obs}$ with unknown observation time. Both $i$ and $j$ represent random subjects [2].

# Supplementary Figure

## Risk Group Distribution


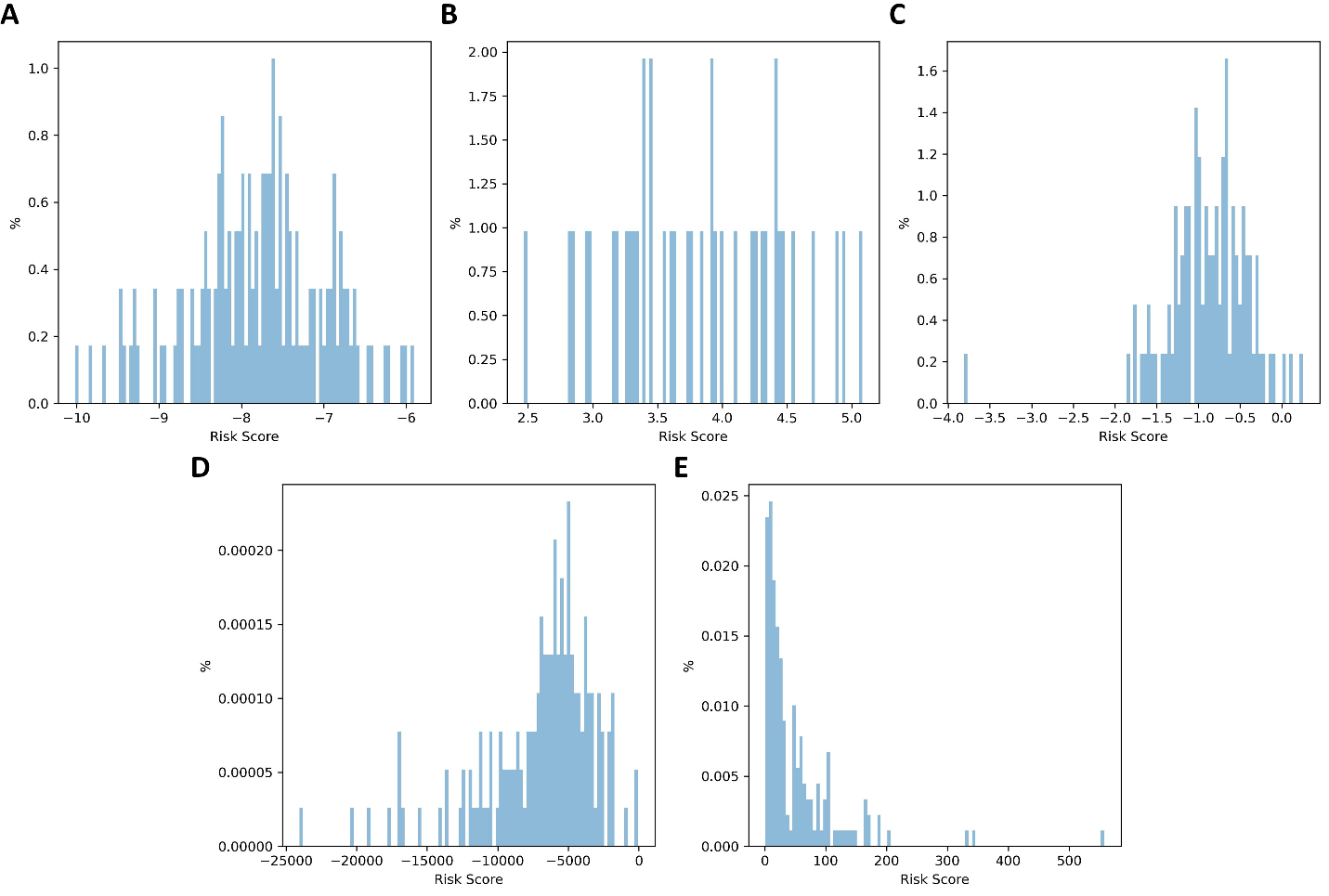


Figure S1. Distribution of risk scores for risk models built on multi-omics data. [(A) and (D)] Transcriptomics baseline model with EV-related genes, (B) Proteomics risk model, (C) DNA methylation risk model, (E) Transcriptomics comparison risk model with all genes. [(D) and (E)] were evaluated on an external dataset, GSE5281 [3–6]. [(A) and (C)] were evaluated on data from ADNI. (B) was evaluated on data from UK Biobank.

# Supplementary Data

**Data S1.** EV-related genes derived from brain tissue samples. All genes were curated from ExoCarta [7]. Malignant tissue samples were disregarded. Five tissue types were used: cortical neurons, microglia, mov neuroglial cells, neural stem cells, and oligodendrocytes.

# Supplementary References

[1] Aryee MJ, Jaffe AE, Corrada-Bravo H, Ladd-Acosta C, Feinberg AP, Hansen KD, Irizarry RA. Minfi: a flexible and comprehensive Bioconductor package for the analysis of Infinium DNA methylation microarrays. Bioinformatics. 2014;30(10):1363–1369. doi:10.1093/bioinformatics/btu049

[2] Longato E, Vettoretti M, Di Camillo B. A practical perspective on the concordance index for the evaluation and selection of prognostic time-to-event models. Journal of Biomedical Informatics. 2020;108:103496. doi:10.1016/j.jbi.2020.103496

[3] Liang WS, Dunckley T, Beach TG, Grover A, Mastroeni D, Walker DG, Caselli RJ, Kukull WA, McKeel D, Morris JC, et al. Gene expression profiles in anatomically and functionally distinct regions of the normal aged human brain. Physiol Genomics. 2007;28(3):311–322. doi:10.1152/physiolgenomics.00208.2006

[4] Liang WS, Dunckley T, Beach TG, Grover A, Mastroeni D, Ramsey K, Caselli RJ, Kukull WA, McKeel D, Morris JC, et al. Altered neuronal gene expression in brain regions differentially affected by Alzheimer’s disease: a reference data set. Physiol Genomics. 2008;33(2):240–256. doi:10.1152/physiolgenomics.00242.2007

[5] Liang WS, Reiman EM, Valla J, Dunckley T, Beach TG, Grover A, Niedzielko TL, Schneider LE, Mastroeni D, Caselli R, et al. Alzheimer’s disease is associated with reduced expression of energy metabolism genes in posterior cingulate neurons. Proc Natl Acad Sci U S A. 2008;105(11):4441–4446. doi:10.1073/pnas.0709259105

[6] Readhead B, Haure-Mirande J-V, Funk CC, Richards MA, Shannon P, Haroutunian V, Sano M, Liang WS, Beckmann ND, Price ND, et al. Multiscale Analysis of Independent Alzheimer’s Cohorts Finds Disruption of Molecular, Genetic, and Clinical Networks by Human Herpesvirus. Neuron. 2018;99(1):64-82.e7. doi:10.1016/j.neuron.2018.05.023

[7] Keerthikumar S, Chisanga D, Ariyaratne D, Al Saffar H, Anand S, Zhao K, Samuel M, Pathan M, Jois M, Chilamkurti N, et al. ExoCarta: A Web-Based Compendium of Exosomal Cargo. Journal of Molecular Biology. 2016;428(4):688–692. doi:10.1016/j.jmb.2015.09.019
